# A Clarion Call for Change: The MLP Imperative to Center Racial Discrimination and Structural Health Inequities

**DOI:** 10.1017/jme.2023.153

**Published:** 2023

**Authors:** Dayna Bowen Matthew, Emily A. Benfer

**Affiliations:** 1. GEORGE WASHINGTON UNIVERSITY LAW SCHOOL, WASHINGTON, DC, USA; 2. PRINCETON UNIVERSITY EVICTION LAB, PRINCETON, NJ, USA

**Keywords:** Public Health, Medical-Legal Partnership, Social Determinants of Health, Discrimination, Racism, Structural Inequities, Health Equity

## Abstract

Across the country, legal and health care professionals who understand that health outcomes are most influenced by social and environmental conditions have improved patient health by adopting the interdisciplinary MLP health care delivery model. However, the MLP field cannot advance population health, let alone long-term health equity, until it addresses the structural determinants of health inequity that are rooted in discrimination, segregation, and other forms of racial and ethnic subordination.

## Introduction

With over 450 sites across forty-nine states and the District of Columbia, the medical-legal partnership (MLP) field has made major strides towards achieving the initial goal of an integrated health care system that leverages social and legal services to achieve positive health outcomes among low-income patients.^1^ Across the country, legal and health care professionals who understand that health outcomes are most influenced by social and environmental conditions have embraced the model and changed the practice of medicine permanently.^2^ As one MLP provider stated, “medical-legal partnership puts the humanity back in medicine…[it] changes the way doctors practice medicine for the rest of their lives.”^3^

To treat the whole patient, hundreds of health care and legal providers have formed MLPs, integrating lawyers and law students into the medical team in nearly all health care settings — from federally qualified health centers and hospital systems to telehealth and specialty practices — to serve the most vulnerable patient populations, including children and infants, elderly people, cancer patients, immigrants, veterans, and formerly incarcerated individuals, among others.^4^ The positive results are widespread and significant: hospital admission rates are reduced among people with chronic illness;^5^ patients experience less stress and improved mental health outcomes;^6^ and emergency department visits decrease.^7^ The use of preventative health care increases, resulting in a reduction of health care spending on high-need, high-cost patients.^8^ In addition, health care costs are reduced, and health care and clinical services are more frequently reimbursed by public and private payers.^9^ Patients also report experiencing improvements in housing and utility needs (82%), personal and family stability needs (73%), education and employment needs (53%), as well as reduced stress (79%).^10^ The model’s success is further amplified in support from the U.S. Department of Justice and Legal Services Corporation, as well as resolutions from the American Bar Association, American Medical Association, and the American Academy of Pediatrics that encourage and promote the development of MLP to “identify and resolve diverse legal issues that affect patients’ health and well-being,”^11^

Despite these outcomes and the rapid growth of the collaborative and interdisciplinary health care delivery model, the MLP field will not and cannot achieve population health, let alone long-term health equity, until it mobilizes and broadens partnerships to address the structural determinants of health inequity that are rooted in institutional racial discrimination, segregation, and implicit bias, as well as other forms of subordination. This article draws from the Supreme Court dissenting opinions in *Students for Fair Admissions, Inc. v. President and Fellows of Harvard College*, together with *Students for Fair Admissions, Inc. v. Univ. of North Carolina*, as legal epidemiology to describe the harm of overlooking the structural determinants of health, specifically racial discrimination.

Despite these outcomes and the rapid growth of the collaborative and interdisciplinary health care delivery model, the MLP field will not and cannot achieve population health, let alone long-term health equity, until it mobilizes and broadens partnerships to address the structural determinants of health inequity that are rooted in institutional racial discrimination, segregation, and implicit bias, as well as other forms of subordination. This article draws from the Supreme Court dissenting opinions in *Students for Fair Admissions, Inc. v. President and Fellows of Harvard College*, together with *Students for Fair Admissions, Inc. v. Univ. of North Carolina*, as legal epidemiology to describe the harm of overlooking the structural determinants of health, specifically racial discrimination. The court’s clarion call underscores the urgent need for the MLP field to address the underlying structural determinants of health that are memorialized in U.S. law and policy and seen all too clearly in health disparities among low-income and historically marginalized MLP patients and communities. With MLPs firmly established in hundreds of hospitals, health centers, legal services organizations and law schools, the national MLP movement is now called to commit to anti-racism and adopt as a core goal the elimination of structural discrimination.

### I. I-HELP and the Structural Determinants of Health Inequity

**Figure 1.**
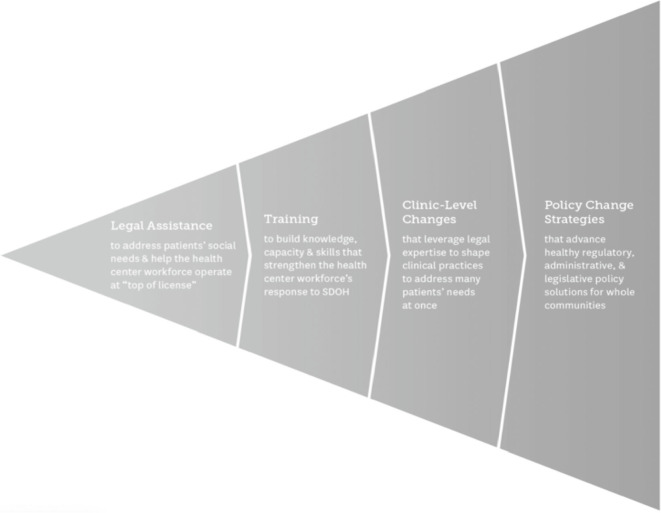
Core MLP Activities, National Center for Medical-Legal Partnership. K. Marple et al., *Bringing Lawyers onto The Health Center Care Team To Promote Patient and Community Health*, National Center for Medical Legal Partnership (October2020): at 4 (reprinted with permission).

The social determinants of health include two critical mechanisms that impact health and well-being: structural determinants of health inequity and intermediary determinants of health.^12^ The structural determinants include the socioeconomic and political context that result in discriminatory policies and other vehicles of subordination.^13^ The structural determinants influence the intermediary determinants, such as employment, housing, food access, and health care. From its beginning, the MLP field set out to address population health problems caused by both structural and intermediary determinants of health on individual and societal levels. The National Center for Medical-Legal Partnership (NCMLP) promotes a flexible four-fold approach to achieve patient and community health: 1) train providers to screen vulnerable patients for health-harming legal needs; 2) treat patients with direct legal assistance that can prevent the most intractable social determinants of poor health; 3) transform clinic practice; and 4) improve population health through joint policy advocacy that leverages MLP knowledge and expertise (See Figure 1).^14^ NCMLP encourages MLPs to engage where they have resources and abilities and to build out partnerships to include partners who can support activities in additional prongs.

In practice, MLPs frequently focus their activities on the first prong. Healthcare providers are trained to screen, identify, and refer individual patients with legal needs that cannot be resolved through medical care alone. Typical screenings inquire into the intermediary determinants of health as reflected in the I-HELP mnemonic: Income, Housing & Utilities, Education & Employment, or Legal Status, Personal and Family Stability (See Table 1).^15^ I-HELP was developed to help lawyers translate civil legal services into health care talking points on how increased access to income, food, and housing, among other benefits, improves patient health and health care delivery. Partners effectively use I-HELP as a guide to MLP coverage areas and as a patient screening tool that has supported the MLP field in effectively transforming clinic practice to identify and treat individual patients with immediate health harming legal needs.

Notably, the I-HELP model has focused primarily on individualized legal services and the impact they can have on a single patient’s life and health outcomes. As important as these impacts are, they fall short of the full range of activities contemplated by the four-fold approach to the medical-legal partnership model as they are insufficient to influence health issues caused by structural problems. The I-HELP categories should also be used to address the structural determinants of health inequity that influence access to each I-HELP area. Left unaddressed, the structural determinants of health inequity limit the movement’s effectiveness and impact, as well as the sustainability of legal interventions.^16^ Namely, racial discrimination and disparities evident in laws, policies, and practices governing each of the I-HELP areas thwart the MLP field’s efforts to achieve widespread health equity. When this discrimination persists at the population level, they produce structural inequalities; when they derive from discrimination and biases that disproportionately affect racial minorities, they are examples of structural racism. The distinction becomes clear when considering the health problem of lead contamination in children. An MLP that compels lead hazard remediation in an individual tenant’s unit to prevent further harm to a lead poisoned child will not achieve health equity in housing unless the underlying laws and policies that were initially rooted in racial bias and that allowed for the harm are addressed.^17^ Moreover, health equity goals will remain elusive until the persistent, disproportionately higher risk of exposure to elevated blood lead levels that non-Hispanic Black children continue to suffer today compared to all other children is also addressed.^18^ The achievement of health equity would require expanding partnerships and increasing resources to allow for the work of amending local building and public health codes to require proactive lead hazard inspections and enforcement of lead hazard abatement orders to undo the harm of legacy lead; addressing disparities in community access to and income to purchase nutritious foods to support the poisoned child’s recovery; collaborating with low-income and historically marginalized communities most exposed to lead hazards to increase awareness and political power; and tackling the structures that allow for disproportionate educational supports by race and socioeconomic status, among other interventions.^19^ However, even these steps are incomplete solutions without also aggressively challenging Fair Housing Act and Title VI violations to address the housing segregation that continues to isolate Black and other marginalized communities from resources while simultaneously ensuring their children’s disproportionate proximity to lead and other environmental toxins.^20^

**Table 1:**
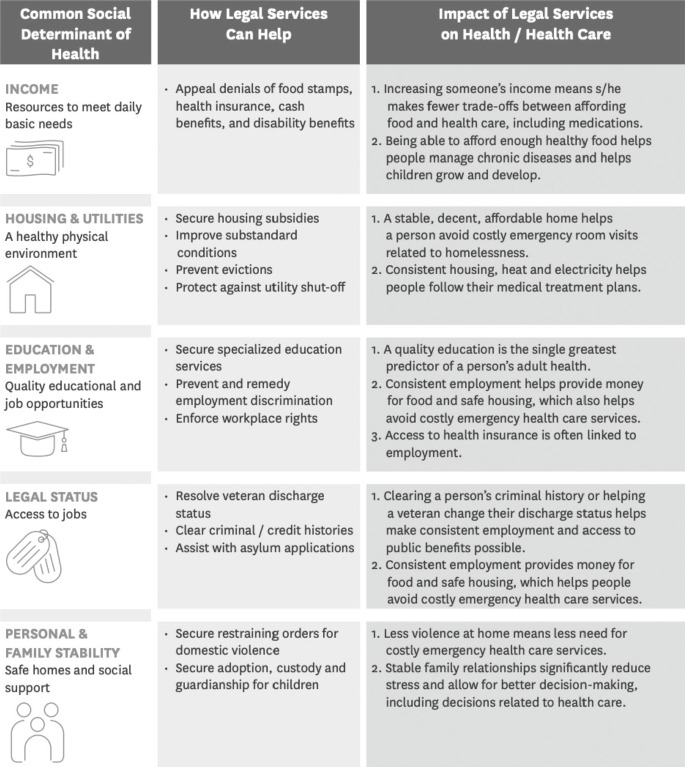
I-HELP: How Legal Services Help Health Care Address the Social Determinants of Health

K. Marple, *Framing Legal Care as Health Care* (Washington, DC: The National Center for Medical Legal Partnership, 2015) (reprinted with permission).

Multiple MLP practitioners and health justice scholars have laid out the importance of centering anti-racism, collaborating with marginalized communities, and addressing the structural determinants of health inequity as the next MLP frontier.^21^ We seek to amplify and expand the urgent call: MLPs, legal institutions, and healthcare systems — as both witnesses to the devastating and life-threatening health effects of racism and experts in the structural and intermediary determinants of poor health, the legal systems that perpetuate harm, and their solutions — must stand as leaders in the effort. We recognize that this vital work is not without barriers. The elimination of structural determinants of health inequities and centering race in MLP work will require an exponential increase in resources, expansion of partnerships, nationwide mobilization led by the National Center for Medical-Legal Partnership, and widespread education of MLP stakeholders on the importance of addressing structural racism to the achievement of health equity. To be clear, it is not solely the responsibility of MLPs; it is the duty of every individual, system, institution, and government, especially those who benefited from centuries of harm, to marshal resources to redress the harmful effects of racism and to adopt laws and policies that both address historic harms and secure future health equity. MLPs can and should be propelling this movement. However, as the legacies of such leaders of the Civil Rights movement such as the Medical Committee for Human Rights,^22^ the National Medical Association,^23^ and the named litigants in the seminal case of *Simkins v. Moses H. Cone Hospital* ^24^ make abundantly clear, only an MLP strategy that unites health care providers, legal experts, and community advocates to confront structural racism can produce transformational societal advances towards true health equity. Yet, today, only a fraction of the MLPs nationwide have formally expanded partnerships and built coalitions that would allow for the anti-racist work of dismantling the inequities within structural determinants of health that plague entire patient communities and perpetuate poor health.

### II. The Health Consequences of Structural Racism in Law and Policy

In their recent dissents from the majority opinion in *Students for Fair Admissions, Inc. v. President and Fellows of Harvard College* and *Students for Fair Admissions, Inc. v. Univ. of North Carolina*, Associate Supreme Court Justices Ketanji Brown Jackson, Sonia Sotomayor, and Elena Kagan reveal the dual tragedy of this omission, first declaring the majority’s disruption of America’s progress toward the Equal Protection Clause promise of educational equality under law “truly a tragedy for us all,” and second identifying the health-harming impact of unchecked racial discrimination for the past four hundred years.^25^ The silver lining, if there is one, is that these three justices have laid out a roadmap to guide MLPs into the next, and perhaps most impactful stage of MLP history: to take up the challenge to address racial discrimination in the structural determinants of health as the persistent and pernicious legal health harm that it is today.

The Justices’ primary purpose was to decry the history and legacy of “state-sponsored race-based preferences in America” that have produced an “intergenerational transmission of inequality that still plagues our citizenry.”^26^ In so doing, they provided ample, constitutionally sufficient justification for the affirmative action programs the majority struck down. But they also did much more than that. While their dissents relied upon legal history, precedent, and an explication of the Constitution’s text, these dissents must also be read as excellent examples of legal epidemiology because they recount how American laws and legal practices that undergird racial discrimination in this country have produced inequitable public health outcomes.^27^ In short, Justices Jackson, Sotomayor, and Kagan painstakingly described the health-harming impact of racial discrimination in this country. They narrated 400 years of discrimination and the modern-day, sequela of health-harming legal needs this history has produced.^28^ Because we know of no MLP that has chosen to address the health-harms of racial discrimination as its mission, we believe the time is ripe for the dissenting opinions in *SFFA v. Harvard and UNC* to be read as a clarion call to the MLP movement to address racial discrimination as the single most important health-harming legal need experienced by minoritized populations in America. To answer this call, MLPs have only to follow Justice Jackson’s dissent which traces the racially discriminatory underpinnings of each of the health harming legal needs that arise within the I-HELP model.

#### A. Income

Justice Jackson began with income inequality. Racially discriminatory laws today include racially disparate tax treatment, the disproportionate location of toxic waste facilities that depress home values in Black communities, and the deliberate location of interstate highways that segregate Black urban poverty.^29^ These health-harming laws are built upon a legacy of laws and legal practices that historically have impeded income and wealth accumulation by Black people. These include legal prohibitions that forbade the sale of land to Freedmen; biases against Black farmers in land giveaways under the Homestead Act from 1862 to 1988; the Federal and state government’s exclusion of Black people from Federal Home Owners’ Loan Corporation subsidies; the Federal Housing Administration’s extreme disproportionate delivery of backed mortgages between 1934 and 1968 to White Americans (98%); and the G.I Bill’s $95 billion gift offered between 1994 and 1971 predominately to White families.^30^ The result, Justice Jackson concludes, is widespread income inequality — the first health harming legal need under the “I-Help” model:

Start with wealth and income. Just four years ago, in 2019, Black families’ median wealth was approximately $24,000. For White families, that number was approximately eight times as much (about $188,000). These wealth disparities ‘exis[t] at every income and education level,’ so ‘[o]n average, white families with college degrees have over $300,000 more wealth than black families with college degrees.’ This disparity has also accelerated over time — from a roughly $40,000 gap between White and Black household median net worth in 1993 to a roughly $135,000 gap in 2019. Median income numbers from 2019 tell the same story: $76,057 for White households, $97,174 for Asian households, $56,113 for Latino households, and $45,438 for Black households.^31^

#### B. Housing

Justice Jackson moved next to the inevitable gaps in home ownership that followed state-sponsored income disparities. She cites studies showing that Black home ownership rates are 25 percentage points behind White home ownership rates and while they tend to be worth less, are subject to higher effective property taxes.^32^ Personally mediated racism both reinforces and is justified by structural racism,^33^ which is exemplified by housing discrimination, insecurity, eviction, serial eviction filing, and substandard conditions that overwhelmingly plague Black families.

#### C. Education and Employment

The discrimination that has produced educational disparities was at the core of the dissenting Justices’ decisions. On this social determinant of health and third prong of I-HELP, Justice Sotomayor’s opinion is particularly poignant. She begins:

Equal educational opportunity is a prerequisite to achieving racial equality in our nation…. Because a foundational pillar of slavery was the racist notion that Black people are a subordinate class with intellectual inferiority, Southern States sought to ensure slavery’s longevity by prohibiting the education of Black people, whether enslaved or free…. Thus, from this nation’s birth, the freedom to learn was neither colorblind nor equal.^34^

This dissenting opinion recounted the Reconstruction Era’s 1866 Freedmen’s Bureau Acts, which were designed to fund black education following the Civil War,^35^ and invoked the opinions of Justice Thurgood Marshall to conclude that history makes it “inconceivable” that race-conscious college admissions are unconstitutional.^36^ Rather, separate educational facilities are inherently unequal and unconstitutional as the Court found in *Brown v. Board of Education*.^37^ Yet these inequities persist today, without any meaningful MLP attention to the discriminatory laws that sustain them, despite evidence of poorer quality facilities in low-income communities of color, disparities in grade level reading proficiency by race, and the disproportionate representation of Black children in special education, expulsions, and suspensions compared to their White peers.^38^ Justice Sotomayor’s dissent recited the data that makes abundantly clear that the problem of school segregation persists today:

After more than a century of government policies enforcing racial segregation by law, society remains highly segregated. About half of all Latino and Black students attend a racially homogeneous school with at least 75% minority student enrollment. The share of intensely segregated minority schools (i.e. schools that enroll 90% to 100% racial minorities) has sharply increased. To this day, the U.S. Department of Justice continues to enter into desegregation decrees with schools that have failed to ‘eliminat[e] the vestiges of *de jure* segregation.’

Moreover, underrepresented minority students are more likely to live in poverty and attend schools with a high concentration of poverty. When combined with residential segregation and school funding systems that rely heavily on local property taxes, this leads to racial minority students attending schools with fewer resources… (noting school funding disparities that result from local property taxation.)

In turn, underrepresented minorities are more likely to attend schools with less qualified teachers, less challenging curricula, lower standardized test scores, and fewer extracurricular activities and advanced placement courses. It is thus unsurprising that there are achievement gaps along racial lines, even after controlling for income differences.^39^

There is a dire and urgent need for MLPs — experts in education law and its health consequences — to respond to the health harming impact of the $23 billion gap that separates state and federal funding for predominately White school districts and funding for predominately Black and Latino districts.^40^ The laws and policies that tolerate discriminatory educational practices that expel and suspend minority students while providing behavioral health supports for White students have largely gone unchallenged, despite their positive relationship to excessive absenteeism, and virtually guarantee the racially disparate educational attainment that is a proximate cause of health disparities.^41^ The connection between discrimination in education, employment, and health outcomes was not lost on the dissenting Justices, however. They described the resulting legal status of minoritized populations by pointing to data that reveals stark racial disparities in unemployment rates, employment practices, consumer transactions, and economic life.^42^ Justice Jackson referenced the discriminatory use of facially race neutral tax and consumer credit laws that keep these disparities in place.^43^

Related to educational barriers, employment equity also remains a significant under-addressed barrier to health. Between 1980 and 2010, workers at the bottom 90% of the workforce realized annual earnings gains of only 15%.^44^ According to the Economic Policy Institute’s low-wage workforce tracker, in 2023, 19.5 million American workers still earn less than $15 per hour, with 18% of Black and Hispanic workers under this threshold, compared to 12% of White workers.^45^

#### D. Personal and Family Instability

Finally, both Justices’ dissents addressed the final element of the I-HELP model, pointing to data that reveal our legal system today produces racialized personal and family instability. Minority children are less likely to have college-educated parents or parents familiar with college application processes.^46^ Their parents are less likely to have access to preschool and other early childhood education programs, and school disciplinary disparities increase the risk of each child’s involvement with the criminal justice system.^47^ Most importantly from a public health perspective, the dissenting Justices recognized that the I-HELP data they referenced represent a group of “interlocked factors [that not only] place underrepresented minorities multiple steps behind the starting line in the race for college admissions,” but also greatly affect minority health and healthcare.^48^ In turn, medically underserved communities experience improved health care access and outcomes when the medical workforce is diverse.^49^ Justice Jackson’s opinion is illustrative:

Health gaps track financial ones. When tested, Black children have blood lead levels that are twice the rate of White children — ‘irreversible’ contamination working irremediable harm on developing brains. Black (and Latino) children with heart conditions are more likely to die than their White counterparts. Race-linked mortality -rate-disparity has also persisted, and is highest among infants.

So too, for adults: Black men are twice as likely to die from prostate cancer as White men and have lower 5-year cancer survival rates. Uterine cancer has spiked in recent years among all women — but has spiked highest for Black women, who die of uterine cancer at nearly twice the rate of ‘any other racial or ethnic group. Black mothers are up to four times more likely than White mothers to die as a result of childbirth. And COVID killed Black Americans at higher rates than White Americans.^50^

While these data are likely familiar to every MLP legal champion across the country, it is important to note the opportunity that stems from the fact that they are being cited in a Supreme Court dissenting opinion: This detailed focus at the highest level of the judiciary drives home the urgent need for a legal response. Three Justices of the United States Supreme Court have causally linked structural inequities to demonstrate the health harming impact of racial discrimination, concluding:

Across the board, Black Americans experience the highest rates of obesity, hypertension, maternal mortality, infant mortality, stroke, and asthma. These and other disparities — the predictable result of opportunity disparities —lead to at least 50,000 excess deaths a year for Black Americans vis-à-vis White Americans. That is 80 million excess years of life lost from just 1999 through 2020.^51^

The need for MLPs to commit to targeting racial discrimination and the structural barriers to health equity has also been clearly laid out by another Associate Justice in the *Students for Fair Admissions v. Harvard College* decision. Justice Neil Gorsuch wrote alone to concur with the majority’s decision to reverse 45 years of affirmative action precedent, but on different grounds: not only does the Equal Protection Clause prohibit this practice, he argues, but Title VI of the Civil Rights of Act of 1964 does as well.^52^ Justice Gorsuch’s opinion espouses a view of Title VI jurisprudence that is supported neither by history, nor the legislative intent of the enacting Congress as determined by the Congressional Record, nor precedent. To reach his conclusion, Justice Gorsuch exploits a false equivalency comparing the Title VI prohibition against exclusion, denial of benefits, and subjugation of minorities to race-conscious methods used by institutions seeking to *reverse and correct* the harms of structural racism against minorities.^53^ He simplistically equates the state-sponsored use of race to effectuate discrimination that *erected* the structural barriers that excluded and subjugated minorities for hundreds of years, to use of race to dismantle those barriers. Moreover, he ignores the historical context and legislative history that gave rise to Title VI. That statute was enacted just as the Fourth Circuit’s decision in *Simkins v. Moses H. Cone Hospital* was being appealed to the Supreme Court.^54^ The Congressional Record reveals that legislators viewed the *Simkins* case as a harbinger of repeated and potentially endless litigation to desegregate hospitals that would result in the absence of a nationwide statute to prohibit racial discrimination by recipients of federal financing.^55^ In fact, Title VI not only tolerates race conscious remedies, but was enacted precisely as a race-conscious remedy in its own right. For decades, courts and regulators have construed Title VI to support and enable remedies to race conscious harms.^56^ MLPs must resist Justice Gorsuch’s attempt to invent a new Title VI jurisprudence at all costs, on all fronts, and with the same force of conviction that motivated Dr. George Simkins and his colleagues to press causes in courts, city councils, and administrative agencies until they desegregated the nation’s hospitals and ushered in the seminal Civil Rights Act of 1964. MLPs must aggressively protect the civil rights laws that have begun to close the gaping divide in resources, health, and legal protections that still separates Black other minoritized populations from White people.^57^ These are the laws that Justice Gorsuch’s undoing of the Civil Rights Act of 1964 would decimate. Justice Gorsuch posits the matter is an “uncomplicated” exercise in semantics. When he parses the Title VI language to interpret “on the ground of” to mean “because of” and argues that a “clear rule emerges” he ignores that the plain meaning of “excluded from participation in, be denied the benefits of, or be subjected to discrimination” referred to massive exclusion of entire racial groups rather than an individual who lost the admission lottery to another candidate on a host of non-racial grounds.^58^ Justice Gorsuch’s analysis focuses on admissions transactions that only barely include considerations of an individual’s *race*, rather than on structural and institutionalized systems that rest wholly on the power of one group of people to exercise their authority over another group of people in the form of *racism*.^59^ In fact, the rule emerging from Justice Gorsuch’s ahistorical semantics may be simplistic but is anything but clear. His view is a grievous obfuscation of the purpose that Congress intended for Title VI: to fight against discrimination “on the ground of” racism so pervasive that it excluded Black patients from White hospitals; Black children from schools; Black taxpayers from voting; and even Black corpses from being buried next to White ones.^60^ In summary, Justice Gorsuch is guilty of mistakenly focusing his Title VI analysis on individual level discrimination, while ignoring the Act’s power to redress structural discrimination that affects all major institutions and systems of our society. This article seeks to steer the MLP community away from a parallel mistake: focusing solely on individual level harm while ignoring the model’s power to effectuate system and community-wide health equity. Justice Jackson characterized the majority’ opinion in *SFFA v. Harvard* as arising from “let-them-eat-cake obliviousness.”^61^ Perhaps even worse, Justice Gorsuch’s decision to turn a blind eye to the dire need today for Black people and other marginalized groups to have equal access to higher education, the weapon of choice against racism that Title VI guarantees, flatly disregards the *imago Dei* in all peoples who live under this nation’s rule of law.^62^

Today’s MLP imperative is to strategically expand the scope of each partnership’s legal work to meet the urgent need to address racial discrimination and the structural determinants of health inequity that perpetuate patient need in the I-HELP areas and result in life-altering health consequences. As Justice Jackson makes clear: “The only way out of this morass — for all of us — is to stare at racial disparity unblinkingly, and then do what evidence and experts tell us is required to level the playing field and march forward together, collectively striving to achieve true equality for all Americans.”^63^

### III. The Clarion Call: MLPs Must Confront Racial Discrimination and the Structural Determinants of Health Inequity

Three crucial takeaways from the dissenting opinions compel the attention of the MLP community. First, the problem of racial discrimination in education, income, housing, employment, health care, the environment, and the other I-HELP areas is the life-or-death, premiere public health crisis of our time. The Centers for Disease Control and Prevention declared racism a public health emergency which demands immediate action.^64^ In addition to the devastating impact of racism in all forms on health outcomes, it is also extremely costly: A recent NIH study determined that the economic burden of health inequities for racial and ethnic minority populations alone is $421-450 billion (or 2% of GDP), stemming from exposure to segregated and disadvantaged neighborhoods, structural discrimination, and racism.^65^ We must locate the crisis squarely within the structural determinants of health inequity and set our sights on changing the social and public laws and policies that are not actively dismantling racial discrimination and advancing health and well-being. Otherwise, the legacy of racism and subordination that currently permeates our legal and social systems will continue to inequitably distribute legal access and material supports under each of the I-HELP categories and further deteriorate the health and well-being of low-income and historically marginalized communities.

Second, the nexus that unifies state-sponsored racial and ethnic discrimination and egregiously disparate health outcomes must become a responsibility that the MLP field also understands as its own. Since the inception of MLP, medical and legal partners have described and documented the effect of discrimination in each of the I-HELP categories in their patients’ lives. The health care industry has labored to address health disparities since the National Academies of Medicine’s consensus report, Unequal Treatment, was published in 2003, dedicating funding to health disparities research and anti-bias training; hiring health equity directors and community organizers; and integrating housing, food supply, and anti-crime services into the health care delivery model.^66^ Community health centers, which emerged from the civil rights movement, have embedded anti-racism into their core activities and dedicated their work to addressing the social determinants of health that have affected Black, Latino, and Indigenous people in areas such as policing, education, employment, housing, safety, and access to health care.^67^ There has been no more urgent time for the MLP field to adopt anti-racism and tackle the structural determinants of health inequity as among its core objectives to counter what appears to be a systematic erosion of the signature enactments of the Civil Rights era.

Third, the MLP model is among the most suitable collaborative techniques for addressing the health-harming problem of racial discrimination within the structural determinants of health. From its inception, the theory of change that animates the MLP model contemplated a range of MLP activities that focused robustly on policy change strategies to produce healthy solutions for whole communities. This is the broad base of the conceptual triangle where MLPs can have their most lasting systemic impact (See Figure 1). Yet, the majority of MLPs have concentrated their activities at the narrow tip of the triangle, providing much needed, but only individually impactful, legal assistance to patients in the clinical setting. In part, this is due to restrictions on legal services activities. Notably, many MLP legal partners are LSC-funded and bound by Legal Services Corporation Act restrictions that prohibit LSC-funded attorneys from engaging in class actions, organizing, or advocating for policies like school desegregation, redistricting reform, and other interventions that could positively affect public health.^68^ In light of these limitations, it is critical for MLPs to expand and partner with academic, legal, nonprofit, and community-based organizations that can organize, lead policy campaigns, initiate impact litigation, and build the necessary evidence to inform policy development. These big tent partnerships allow MLP providers to not only ask what structural racism-related law, policy, or practice caused their patient’s health-harming legal or social issue, but also who else is needed at the table and what resources are necessary to address its structural and systemic causes. The multi-lens perspective of the interdisciplinary team, combined with community-based collaboration, supports the evidence-gathering needed to demonstrate a pattern of harm, accurate problem statements, and innovative solutions. Ultimately, we agree that no MLP is exempt from this imperative to help realize laws and policies that redress historic harm and provide the legal protection and social supports to achieve health equity.^69^

The solution we propose is a massive expansion in MLP resources and strategies to address laws and policies informed by racial discrimination that are destroying whole generations of historically marginalized communities in America.

The solution we propose is a massive expansion in MLP resources and strategies to address laws and policies informed by racial discrimination that are destroying whole generations of historically marginalized communities in America. It is no longer enough to solely provide I-HELP-based legal services to individuals and families. MLPs must partner with research universities to conduct and disseminate the kind of research that a majority of Supreme Court justices say they need to justify a compelling governmental interest in righting the debilitating racial wrongs that continue to plague entire communities. MLPs must vigorously represent — alone or through firm partnerships — Black and Indigenous communities and communities of color in challenging Health and Human Services, the Department of Housing and Urban Development, and the Environmental Protection Agency to make findings of discrimination so that communities can secure protection against agricultural, industrial, transportation, and other deliberately situated sources of health hazards that disproportionately burden minority families.^70^ MLPs must join with civil rights, children’s rights, environmental and housing justice, and public health groups, among others, to bring litigation that challenges school finance discrimination based on the uncontroverted nexus between a lack of educational attainment and disparate illnesses that end in early deaths.^71^ MLPs must partner with communities and hospitals to map and screen victims of elevated lead levels in soil, paint, dust, and pipes; identify concentrations of building code violations that betray structural housing defects; and attack pervasively discriminatory eviction practices. MLPs must build coalitions that take up legislation and litigation to advance healthy regulatory, administrative, and policy solutions for whole communities. Academic and non-LSC funded MLPs must leverage their patients-to-policy capacity and work in collaboration with legal services-staffed MLPs. MLPs must increasingly partner with public health researchers and social scientists to engage in policy surveillance to identify structural racism-related laws and policies and collaborate with advocates to spur adoption of those policies that will contribute to lasting health equity. Of utmost importance, MLPs must partner with and follow the lead of low-income and historically marginalized communities to identify patterns of discrimination, develop pathways for enforcement of rights, and to inform and implement the laws and policies that will set them on the path to health and well-being. This is just the start.

MLPs have already demonstrated their ability to address our country’s sordid history of racially discriminatory law and policy. To address lead poisoning among patients in federally assisted housing, the Health Justice Project at Erie Family Health Center in Chicago developed a national coalition to amend antiquated federal policy that effectively treated low-income, Black and Latino children like canaries in the coalmine by identifying lead hazards in their developing brains and bodies instead of through scientific tools. The Center for Children’s Advocacy in Connecticut joined a statewide coalition to compel the passage of healthcare for undocumented children. The Health Justice Advocacy Clinic in New York City partnered with the National Housing Law Project to ensure that public housing was held to the same standards as private market homes, compelling the passage of legislation to require carbon monoxide detectors. The Children’s Law Center in Washington, D.C. partnered with Children’s National Hospital to map multi-family buildings that represent the highest rates of asthma-related pediatric emergency department visits and unhealthy housing conditions that contribute to asthma severity. The interactive tool allows the MLP to collaborate with the city to enforce housing code laws for entire communities and before children are forced into acute respiratory distress. MLPs have also leveraged the expertise of their interdisciplinary teams and joined with national public health associations and experts to file successful amicus briefs in federal court to address harmful lead hazard standards, eviction during the COVID-19 pandemic, and gun policy affecting victims of domestic violence. George Washington University Law School launched the Health Equity Policy & Advocacy Clinic, an MLP with Bread for the City focused on addressing the structural determinants of health inequity in housing by applying the health justice framework. In addition to addressing the needs of individual clients, the Health Equity Policy & Advocacy Clinic partners with organizers, community groups, and tenants to identify and address the historic, systemic, and structural racism at the root of substandard housing conditions, high rates of housing instability, and severe health inequity in majority-Black neighborhoods.^72^ The clinic underscores the importance of partnering with the community and the necessity of building the political capacity of people who are disproportionately harmed by health inequity and who must be involved in the development of policies that will affect their lives.^73^

## Conclusion

As the dissenting opinions in *Students for Fair Admissions, Inc.* so clearly laid bare, the structural determinants of health, including racial discrimination, poverty, and other vehicles of subordination and subjugation, threaten the health and livelihood of millions of Americans. The MLP field must shift its focus, and build capacity, to address the longstanding and racially discriminatory underpinnings of poor health among patients. There is no better model than the MLP, with the combined expertise of medical and legal champions, to address discrimination within the structural determinants of health inequity. Especially as the Supreme Court’s threats to dismantle seminal civil rights laws increase and so-called “originalist” theorists ignore the health effects of centuries of discriminatory harms, it is paramount that the MLP movement answer the clarion call to center racism and work collaboratively to eliminate racial discrimination and structural health inequities as the next and only step forward.

## Note

In 2024, Benfer will be assuming a leadership role in the National Center for Medical-Legal Partnership and was not engaged in this role during the manuscript drafting or publication.
